# The Latest Research on Probiotics: Nourishing Health or Pathways to Disease Resolution

**DOI:** 10.3390/nu17010019

**Published:** 2024-12-25

**Authors:** Po-Wen Chen

**Affiliations:** Department of Veterinary Medicine, College of Veterinary Medicine, National Chung Hsing University, Taichung 402202, Taiwan; powenchen@nchu.edu.tw

## 1. Stress and Mental Health

The meta-analysis on probiotics’ effects on cortisol levels demonstrates their potential to modulate stress responses across diverse populations, though variability in strain efficacy highlights the need for standardization and targeted studies. Similarly, a multi-strain E3 probiotic was shown to improve mental health and sleep quality, linking gut microbiota interventions to broader psychological outcomes.

## 2. Disease-Specific Applications

From antidiabetic effects to enhancing vaginal microbiota in postmenopausal women, specific probiotic strains like *Lactiplantibacillus plantarum* and *Bifidobacterium animalis* exhibit targeted health benefits. These studies emphasize the importance of strain-specific interventions tailored to individual or population health needs

## 3. Mechanistic Insights

Advances in understanding mechanisms, such as the role of *L. plantarum* in inducing autophagy and enhancing epithelial barrier function, underline the intersection of probiotics with cellular processes. Investigations into the Muribaculaceae family further enrich the taxonomy of gut microbiota, revealing new metabolic pathways.

## 4. Prebiotic Innovations

Lactoferrin’s dual direct and indirect prebiotic effects open pathways for its application in disease control, showcasing the potential of combining probiotics with advanced prebiotics to enhance gut health synergistically.

## 5. Dietary Integrations

Exploring vegan probiotic mixtures highlights the feasibility of combining probiotics with plant-based diets to maintain viability during digestion and improve bioactive compound accessibility.

## 6. Addressing Knowledge Gaps

- Efficacy Variability: The diverse outcomes in cortisol reduction and disease-specific benefits indicate a need for more personalized and context-specific probiotic formulations.
- Mechanistic Studies: While emerging evidence links microbiota modulation to outcomes like improved brain-derived neurotrophic factor (BDNF) levels or barrier integrity, further exploration of molecular pathways is required.
- Multi-Omics Integration: Existing research could be expanded with proteomics and metabolomics to better link microbiota composition with systemic effects.

## 7. Future Research Objectives

1. Standardization and Personalized Probiotics: Standardizing probiotic strain selection and identifying biomarkers for individual responses will enhance clinical applications.
2. Expanded Applications: Investigating probiotics in underexplored areas, such as metabolic flexibility or rare conditions like polymicrobial infections.
3. Combination Therapies: Studies on combining probiotics with nutraceuticals, prebiotics, or pharmaceuticals to achieve synergistic effects.
4. Global Diversity: Research should include diverse populations to address geographical and dietary influences on probiotic efficacy.
5. Sustainability in Probiotics: Exploring the production of vegan or bioengineered probiotic solutions to align with environmental sustainability.

This Special Issue sheds light on the multifaceted roles of probiotics and prebiotics in health, paving the way for targeted, sustainable, and innovative interventions.

